# Markov State Models and Molecular Dynamics Simulations Provide Understanding of the Nucleotide-Dependent Dimerization-Based Activation of LRRK2 ROC Domain

**DOI:** 10.3390/molecules26185647

**Published:** 2021-09-17

**Authors:** Xinyi Li, Zengxin Qi, Duan Ni, Shaoyong Lu, Liang Chen, Xiangyu Chen

**Affiliations:** 1 School of Medical Laboratory, Weifang Medical University, Weifang 261053, China; Ashley_Li@sjtu.edu.cn; 2 Medicinal Chemistry and Bioinformatics Center, School of Medicine, Shanghai Jiao Tong University, Shanghai 200025, China; niduan11@sjtu.edu.cn; 3 Department of Neurosurgery, Huashan Hospital, Fudan University, Shanghai 200040, China; qizengxin@huashan.org.cn; 4 Shanghai Key Laboratory of Brain Function Restoration and Neural Regeneration, Huashan Hospital, Fudan University, Shanghai 200040, China; 5 State Key Laboratory of Medical Neurobiology and MOE Frontiers Center for Brain Science, School of Basic Medical Sciences and Institutes of Brain Science, Fudan University, Shanghai 200433, China

**Keywords:** Parkinson’s disease, leucine-rich repeat kinase 2 (LRRK2), Ras-of-complex GTPase domain, molecular dynamics (MD) simulations, Markov state models, network analysis

## Abstract

Mutations in leucine-rich repeat kinase 2 (LRRK2) are recognized as the most frequent cause of Parkinson’s disease (PD). As a multidomain ROCO protein, LRRK2 is characterized by the presence of both a Ras-of-complex (ROC) GTPase domain and a kinase domain connected through the C-terminal of an ROC domain (COR). The bienzymatic ROC–COR–kinase catalytic triad indicated the potential role of GTPase domain in regulating kinase activity. However, as a functional GTPase, the detailed intrinsic regulation of the ROC activation cycle remains poorly understood. Here, combining extensive molecular dynamics simulations and Markov state models, we disclosed the dynamic structural rearrangement of ROC’s homodimer during nucleotide turnover. Our study revealed the coupling between dimerization extent and nucleotide-binding state, indicating a nucleotide-dependent dimerization-based activation scheme adopted by ROC GTPase. Furthermore, inspired by the well-known R1441C/G/H PD-relevant mutations within the ROC domain, we illuminated the potential allosteric molecular mechanism for its pathogenetic effects through enabling faster interconversion between inactive and active states, thus trapping ROC in a prolonged activated state, while the implicated allostery could provide further guidance for identification of regulatory allosteric pockets on the ROC complex. Our investigations illuminated the thermodynamics and kinetics of ROC homodimer during nucleotide-dependent activation for the first time and provided guidance for further exploiting ROC as therapeutic targets for controlling LRRK2 functionality in PD treatment.

## 1. Introduction

**Figure 1:**
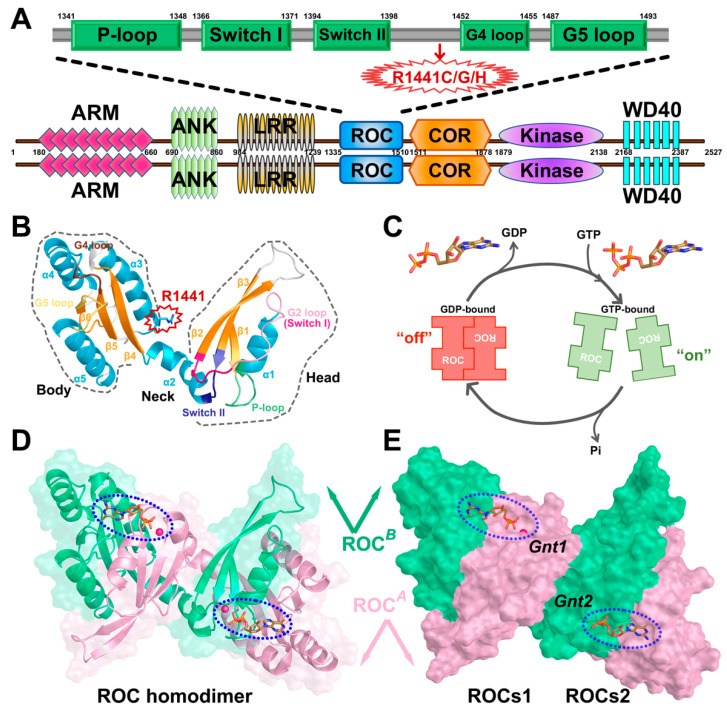
(**A**) Domain architecture of human LRRK2 with its respective amino acid positions. ARM: Armadillo repeats region; ANK: Ankyrin repeat region; LRR: leucine-rich repeats; ROC: Ras-of-complex GTPase domain; COR: C-terminal-of-ROC domain; Kinase: protein tyrosine kinase-like domain; WD40: WD40 repeat. The detailed functional partition of ROC is depicted above with the guanine nucleotide phosphate-binding loop (P-loop), Switch I and Switch II, and G4 and G5 loop highlighted in green rectangles. The residue position R1441 is highlighted with a red arrow. (**B**) Cartoon model of ROC monomer roughly divided into head (including β1, α1, β2, and β3), neck (including α2) and body (including β4, α3, β5, α4, β6, and α5) subdomains. The monomer is colored based on a secondary structure, and the P-loop, Switch I, Switch II, G4 loop, and G5 loop are highlighted in light green, hot pink, purple, brown, and gold, respectively. Residue R1441 is highlighted. (**C**) Schematic model of ROC dimer–monomer dynamic transition during nucleotide turnover based on GAD theory. (**D**) Cartoon model of ROCs-GDP, the two monomers are shown in pink (ROC*^A^*) and green (ROC*^B^*), respectively. (**E**) Molecular surface representation of ROC dimer highlighting the nucleotide-binding pockets (blue-dashed oval). *Gnt1* is accommodated by ROC*^A^* head and ROC*^B^* body, while *Gnt2* interacts with ROC*^A^* body and ROC*^B^* head. Unless otherwise specified, the graphs showing ROCs overview in our work will all be presented from this perspective with *Gnt1* residing on the top-left. The presumed compact functional units are denoted as ROCs1 and ROCs2.

The rapidly ageing population has imposed challenging public health crises in dealing with age-associated neurodegenerative afflictions with effective treatments [1,2,3]. As one of the most prevalent neurodegenerative disorders, Parkinson’s disease (PD) features symptoms including muscular rigidity, resting tremor, and movement slowness [4,5]. Pathologically, accumulating evidence has suggested that progressive degeneration of dopaminergic (DA) neurons and the presence of abnormal intraneuronal cytoplasmic inclusions (termed Lewy bodies) rich in α-synuclein are hallmarks in PD pathogenesis [6,7,8]. Although intense efforts have been made to understand this desperate disease, the etiology of PD remains unresolved, while both environmental and genetic factors are suggested to play a part [9,10,11]. To date, missense mutations in leucine-rich repeat Kinase 2 (LRRK2), the gene product of Parkinson’s recognized risk loci PARK8, have been recognized as the most frequent cause of PD among all relevant pathogenetic genes identified [12,13,14]. LRRK2 belongs to the superfamily of ROCO proteins, a unique feature of which is the presence of a Ras-of-complex domain (ROC) in tandem with a C-terminal of a ROC domain (COR), immediately followed by a tyrosine kinase-like protein kinase domain (kinase) [15,16]. Meanwhile, additional protein–protein interaction domains are also found in the N-terminus (armadillo: ARM, ankyrin: ANK, and leucine-rich repeats: LRR) and C-terminus (WD40 repeats) of the central ROC–COR–kinase catalytic triad of LRRK2 [17,18,19] (Figure 1A). ROC contains five α helices and six β strands connected through loops, and can be roughly divided into head (β1, α1, β2, and β3), neck (α2), and body (β4, α3, β5, α4, β6, and α5) subdomains [20] (Figure 1B). Crystallographic studies have revealed the unique homodimeric structure of ROC, as LRRK2 exists predominantly in the form of dimers in cells and tissues (ROC dimer: ROCs, Figure 1D). With the domain-swapping dimerization scheme, ROCs form two nearly symmetric nucleotide-binding sites (referred to as *Gnt1* and *Gnt2*) with structural motifs contributed from both monomers (referred to as ROC*^A^* and ROC*^B^*, Figure 1E). Moreover, subdomains of each ROC monomer can further pair with another and form compact functional units with corresponding nucleotide-binding sites (ROCs1 and ROCs2, Figure 1E), which resembles the classic scheme adopted by small GTPase [21,22,23]. Of note, the bienzymatic architecture of LRRK2 implies that the GTPase and kinase activity might be coupled via an intramolecular mechanism with GTPase activity serving to regulate kinase activity [24,25,26], and this notion is further supported by the observation that disease-causing missense mutations within the ROC domain, the famous R1441C/G/H point mutations, for instance [27,28,29], usually result in up-regulated kinase activity [14,30]. However, as a functional GTPase, the detailed mode of action of ROC remains elusive, but studies on LRRK2 homologs from prokaryotes have suggested that LRRK2 ROC is likely to function as a G protein activated by nucleotide-dependent dimerization (GAD) [31], which relies mainly on spontaneous nucleotide turnover and dimerization for the regulation of the activation cycle [32,33] (Figure 1C). Under this circumstance, exploration of the conformational dynamics relevant to the dimerization and nucleotide turnover of LRRK2 ROC is of primary importance for further investigations into its contribution to the overall functional output of LRRK2 under both physiological and pathological conditions.

Nonetheless, with the existing experimental methodologies, it remains challenging to describe the dynamic conformational transition process, especially in the case of binding ligand turnover, which usually results in distinctive structures with different bioactivity [19,34,35,36,37]. To obtain a comprehensive insight into the dynamics of ROC homodimer during nucleotide shift, we employed large-scale all-atom molecular dynamics (MD) simulation to observe in silico at the atomistic level the intrinsic regulation of GTPase activation cycle of the ROC domain of LRRK2. Furthermore, by integrating our extensive MD simulations with the statistically robust Markov state models (MSMs) for interpreting configuration sampling [38,39,40], kinetically relevant states of ROCs homodimer as well as their interconversion rates during GDP/GTP exchange are accessible. Based on MSMs, our study provided for the first time the dynamic portrait of ROCs during nucleotide turnover, as characterized by the previously unreported coupling between dimerization and nucleotide-binding state, with the ROCs dimer exhibiting the “open” conformation with greater dimerization extent in the presence of GDP but switching to the “closed” structure with oligomerization tendency while in complex with GTP. Meanwhile, through dynamic network analysis [41], we dissected the intramolecular correlation network mediating such global structural rearrangements. Furthermore, inspired by the disease-causing mutations clustered within ROC domain, we found that such mutants, as exemplified by R1441C/G/H, possess the potential to remarkably accelerate the interconversion between GDP-bound inactive and GTP-bound active states. The global effects introduced by such single-residue substitution indicated potential allosteric regulation, and we thus probed into the allosteric signaling pathway associated with mutations. We demonstrated that signals associated with R1441C/G/H mutation could propagate toward the nucleotide-binding sites of ROCs, and more importantly, we disclosed the α2, β4 of both ROC monomers as relatively conservative components for transmitting such allosteric signals, providing guidance for the identification of potential allosteric pockets. Our studies supplement for the first time a dynamic picture of the LRRK2 ROC domain during nucleotide turnover, which supported the presumption that LRRK2 adopted a nucleotide-dependent dimerization-based activation scheme like that of GADs. In the meantime, we also uncovered the detailed molecular mechanism for the pathogenetic effects of R1441C/G/H mutations, which further led us to the identification of important allosteric signal transducers. The results offer an insightful understanding toward of the GTPase activation cycle of the ROC domain as well as its role in regulating LRRK2 functionality, and such mechanistic insights provide guidance for targeting ROCs for tuning LRRK2 bioactivity for future PD treatment.

## 2. Results

### 2.1. Construction and Validation of Markov State Models (MSMs)

**Figure 2:**
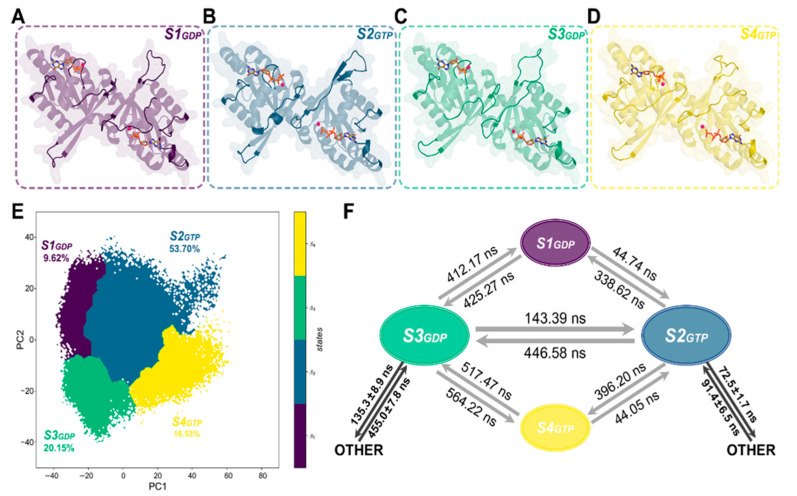
Representative structures of the four metastable states in ROCs-GDP~ROCs-GTP MSMs: (**A**) *S1_GDP_* for macrostate *S1*, (**B**) *S2_GTP_* for macrostate *S2*, (**C**) *S3_GDP_* for macrostate *S3*, and (**D**) *S4_GTP_* for macrostate *S4*. Structural segments that exhibited significant differences are highlighted in the opaque cartoon, with the remaining being transparent. (**E**) Distribution of the four metastable states in Rocs-GDP~ROCs-GTP MSMs with the portion of each macrostate. (**F**) Transition time between the macrostates calculated by mean first passage time (MFPT). OTHER: denoted the transition between *S2_GTP_*/*S3_GDP_* and all other remaining metastable states.

The solved crystal structure of ROC homodimer (ROCs) in association with either guanosine diphosphate (ROCs-GDP, PDB ID: 2ZEJ [20]) or guanosine triphosphate (ROCs-GTP, artificially built by substituting GDP with GTP in ROCs-GDP complex, detailed in Section 4) were subjected to unbiased large-scale classic MD simulations to dissect the dynamic conformational regulation contributed by guanine nucleotide turnover at an atomistic level. With the refined starting crystal structures, we gathered a total of 6 μs extensive sampling with 1 × 3 μs independent runs with random initial velocities for both systems, which permitted more complete and thorough mapping of the conformational landscape of ROCs throughout its activation cycle. Both systems reached equilibrium during 1 μs MD simulation in every replica (Section 4, Figure S1). Furthermore, to identify the key components along the activation pathway, we employed the kinetic network MSM embedded in PyEMMA [42]. The simulation trajectories of both ROCs-GDP and ROCs-GTP were first featurized with inter-residue distances to describe the overall ROCs topology, and the resulting dynamics data were subjected to principal component analysis (PCA) from *scikit-learn* for dimensionality reduction to preserve the first two components (PC1 and PC2) with the greatest contribution to kinetic variance [43]. The Markovianity of our model was validated through implied timescales estimations [44,45] (Figure S2A). A lag time of 3 ns was selected for MSM construction, and 100 microstate centers were chosen for the *k-means* clustering algorithm [46]. The obtained microstates were further clustered into four metastable states (also called macrostates) using the *PCCA+* algorithm with a passing Chapman-Kolmogorov test [47,48] (Figure S3), obtaining the ROCs-GDP~ROCs-GTP MSM that contains the thermodynamics and thermokinetics of ROCs during activation (Figure 2E).

### 2.2. MSMs Revealed Different Dimerization Extent of ROCs during Nucleotide Turnover

Based on our constructed ROCs-GDP~ROCs-GTP MSMs, the conformational space of ROCs during guanine nucleotide turnover was divided into four metastable structures as shown in Figure 2A. From the distribution of ROCs-GDP and ROCs-GTP on the 2-dimensional plane defined by the two principal components (Figure S4A, Table S1), metastable structures *S1* and *S3* were found to be dominated by the ROCs-GDP conformational ensemble and more likely to represent the “inactive” resting conformation of ROCs under GDP-bound state. In contrast, a greater population of ROCs were found to bind GTP in macrostates *S2* and *S4*, which might denote the active state. To identify key structural features of ROCs under either GDP- or GTP-bound states, we extracted the representative conformations from the four metastables in their more prominent nucleotide-binding state and obtained *S1_GDP_*, *S2_GTP_*, *S3_GDP_*, and *S4_GTP_* for further investigations (Figure 2A–E, Table S1).

A closer insight onto these representatives unveiled that Switch I, β2–β3 linker, G4 loop, and G5 loop were the primary sites for conformational rearrangements (compare Figure 2A–D with Figure 1B). As the representative structure for ROCs-GDP and ROCs-GTP, *S3_GDP_* and *S2_GTP_* exhibited significantly different structures in the above-mentioned motifs, with *S3_GDP_* forming a relatively more compact conformation as the implicated structural segments were posing closer to the main body (Figure 2B,C), and this suggested that the extent to which ROC monomer dimerized may be dissimilar. Indeed, looking into the extensive contact network between the ROC monomers proved that either the stability or the strength of the interactions formed between monomers to facilitate dimerization weakened prominently upon substituting GDP with GTP (Table S2). In the meantime, the interfacing surface area between monomers also decreased (Table S3), further weakening the intramolecular interactions and destabilizing dimerization. Moreover, by calculating the binding free energy between the ROC monomers with Molecular Mechanics/Poison Boltzmann Surface Area [49,50,51] (MM/PBSA), we further confirmed that ROCs, when bounded by GTP, were indeed less dimerized and therefore more prone to form the catalytically active monomers with hydrolysis activity than to be bound by its GDP counterparts.

Furthermore, we computed the mean first passage time (MFPT) for transition between the dominant conformations *S3_GDP_* and *S2_GTP_* based on the established MSM model (Figure 2F). According to the results, while *S3_GDP_* can directly transit to *S2_GTP_* relatively fast (143.39 ns), the time for transition from *S2_GTP_* to *S3_GDP_* was more than three times the reverse (446.58 ns). Additionally, compared with *S3_GDP_*, *S2_GTP_* can more quickly transit to other states, which could be in line with the transient appearance of the active state during GTPase cycle. The unmatched interconversion rates between ROCs-GDP (*S3_GDP_*) and ROCs-GTP (*S2_GTP_*) observed in our simulation study, as exemplified by the considerably longer time taken from GTP-bound state back to GDP according to MFPT results, hinted that there could be undiscovered GTPase-activating proteins (GAPs) that help with accelerating this transition process under physiological conditions to keep this activation cycle in control.

Taken together, based on the ROCs-GDP~ROCs-GTP MSMs, although there is evidence claiming that ROCs form symmetric homodimers spontaneously, it appears that this process may not be a completely segregated from of nucleotide turnover, as GTP binding indeed induced prominent global conformational rearrangements in key structural motifs including Switch I, G4 loop, G5 loop, etc., while also rendering the whole ROCs complex less dimerized through altering the inter-monomer recognition pattern. In the meantime, the considerably longer time needed for transition from a GTP- to GDP-bound state also suggested the potential presence of GAPs as a catalyst for the ROC activation cycle [14,52].

### 2.3. ROCs Exhibited Classic “Open” to “Closed” Conformational Transition of GTPase

**Figure 3:**
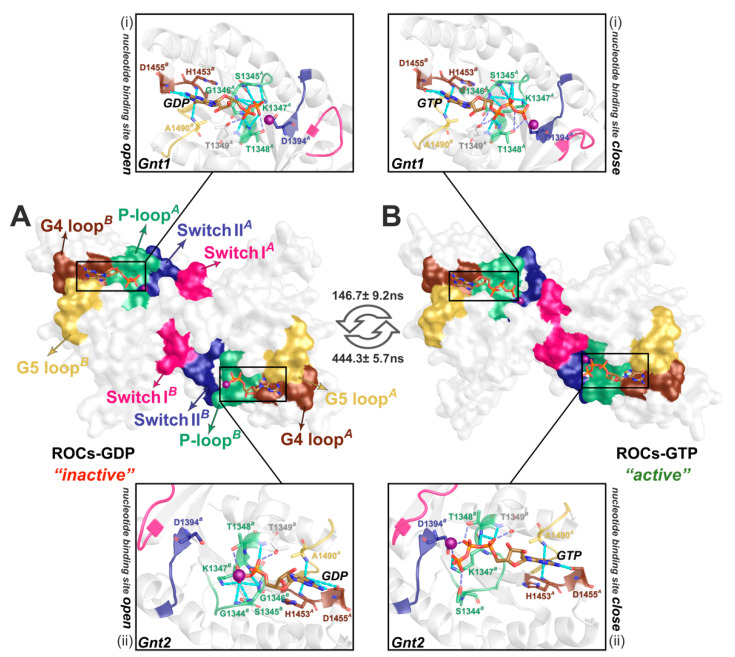
Detailed bonding network between the guanine nucleotide and ROCs in the representative structure from (**A**) ROCs-GDP, *S3_GDP_*, and (**B**) ROCs-GTP, *S2_GTP_*. The molecular surface diagram in the middle highlighted regions implicated in nucleotide binding, as color-coded in Figure 1B. The zoom-in graphs above (i) and below (ii) depicted the detailed interactions between ROCs and nucleotide *Gnt1* and *Gnt2*, respectively.

As the primary function executed by ROCs, the GTPase activity is of interest due to their potential to fine-tune the bioactivity of the whole LRRK2 complex. Derived from the most-representative Ras from the small GTPase superfamily, whose nucleotide turnover typically leads to conformational transitions that is closely coupled with activation state, we also wonder if there would be considerable structural changes in regions contacting guanine nucleotide in the presence or absence of the γ-phosphate. With the identified representative metastable states in ROCs-GDP~ROCs-GTP MSM, *S3_GDP_*, *S2_GTP_* represented the “inactive” and “active” state of ROCs (Figure 3). As previous studies suggested [20], the nucleotide-binding pockets on ROCs are contributed by regions from both monomers, and P-loop, Switch I, and Switch II, together with G4 and G5 loop from the other monomer, have been shown to play a key role (Figure 3). The results implied that the symmetric homodimerization pattern of ROC monomers rendered its two nucleotide-binding pockets indistinguishable in their interactions with GDP/GTP. For *Gnt1*, the guanine head in both GDP and GTP bonded with neighboring H1453*^B^* and D1455*^B^* of G4 loop, and also A1490*^B^* of G5 loop, while the polar residues S1345*^A^*-T1349*^A^* of P-loop interacted with the α- and β-phosphate group of the nucleotide. Of note, this interaction network was stronger in the presence of GTP. Upon elongation of the phosphate tail with a γ-phosphate group by substituting GDP with GTP, T1348*^A^* formed additional interactions with the γ-phosphate, and D1394*^A^* of Switch II also hydrogen-bonded with the γ-phosphate; on the basis of enhanced interaction between GTP and neighboring residues, these together stabilized GTP binding and created a relatively “closed” conformation amenable for GTP hydrolysis (Figure 3B). Similar structural transitions were also observed in motifs mediating *Gnt2* binding. Such changes in regions implicated in nucleotide binding toward a “closed” conformation during the exchange of GDP with GTP in the activation cycle were also observed for classical GTPase including Ras [38], and their similarities could provide guidance on further investigations regarding the detailed GTP hydrolysis process by the ROC homodimer of LRRK2.

### 2.4. Nucleotide Turnover Reshaped ROCs Global Structure through Correlated Network

**Figure 4:**
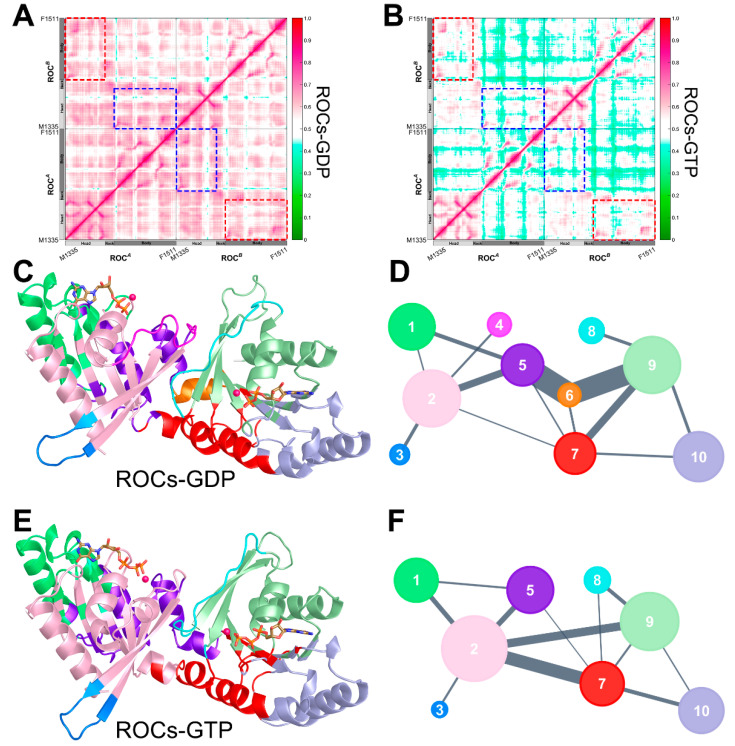
Generalized cross correlation matrix (GCCM) of (**A**) ROCs-GDP and (**B**) ROCs-GTP. The colormap used for the graph is shown on the right with no/weak correlations indicated in green and strong ones in magenta. The correlation between ROC*^A^* head and ROC*^B^* body, and that between ROC*^A^* body and ROC*^B^* head subdomain, is highlighted in red and blue dashed line rectangles, respectively. Cartoon representation of (**C**) ROCs-GDP and (**E**) ROCs-GTP color-coded based on their corresponding community structures. Simplified community network map with inter-community crosstalk in (**D**) ROCs-GDP and (**F**) ROCs-GTP. Each community in the original structure is reduced to a sphere whose area is proportional to the residue components. Inter-community crosstalk is visualized by sticks of width that are proportional to edge connectivity between communities.

Generalized cross-correlation matrix [53] (GCCM), which is based on the fundamental definition of independence of random variables, was a well-established method to quantify both linear and nonlinear correlations and was thus employed to probe into the correlated motions that could be essential for ROCs functionality during the nucleotide shift. GCCM revealed that GTP substitution altered the correlative motion within ROCs (Figure 4A), including both inter- and intra-monomeric correlations. Generally speaking, the overall correlation network was significantly weakened in the presence of GTP, and this net effect was further demonstrated by reduced coupled dynamics between ROC*^A^* and ROC*^B^*, as well as within each monomer (highlighted in rectangles in Figure 4A). It is worth noting that inter-monomeric correlations dropped drastically, with the body subdomain, the primary part to provide an anchor for interactions stabilizing ROC dimerization, of each monomer exhibiting considerably weaker collective motion with the other monomer. Experiments have proposed that subdomains from ROC*^A^* and ROC*^B^* contributed to the formation of functional units ROCs1 and ROCs2 with a guanine nucleotide-binding site facilitated by polar interactions mediated by residues from both monomers. This decrease in inter-monomeric correlations indicated to a certain extent that the interactions between monomers that could be relevant to ROC dimerization were weakened upon substituting GDP with GTP. The observed change in GCCM correlation that mediated communication between distant regions was in line with the “open” to “closed” conformational shift during nucleotide exchange, and the weakened coupled motions between ROC monomers could be associated with the nucleotide-dependent dimerization-based activation of ROCs GTPase.

To investigate how intramolecular signals associated with nucleotide turnover propagate throughout the ROC complex to induce the observed shift in conformational landscape, we further conducted community network analysis (CNA) to illuminate the dynamic signal transduction scheme within ROCs during nucleotide exchange [54,55]. With CNA, through assigning nodes and weighting edges based on GCCM, closely related residues were grouped into inter-connected “communities”. The obtained community substructures for both ROCs-GDP and ROCs-GTP were shown in Figure 4C–F, and it turned out that the overall network partition was altered significantly upon nucleotide shift. In the presence of GDP (Figure 4C,D), the community structure of ROCs was more fragmented, and *Community* #6, consisting predominantly of ROC*^B^* neck residues, served as a hub for transmitting signals between the relatively distant head (*Community* #9) and body (*Community* #5) portion of ROC*^A^*. Meanwhile, strong information flow was also observed between ROC*^B^* head (*Community* #2) and ROC*^A^* body (*Community* #5), as well as ROC*^A^* head (*Community* #9) and ROC*^B^* body (*Community* #7). Additionally, 7 out of 10 communities were found to encompass residues from both monomers (Table S4), demonstrating the considerably strong reciprocal interactions between the two monomers, which we believe to be relevant to dimerization. Upon substitution to GTP (Figure 4E,F), however, the number of communities was reduced to 8, while weaker intercommunications were found between communities. The head (*Community* #2) and body (*Community* #7) subdomain of ROC*^B^* exhibited stronger and more direct connection, and although *Communities* #2, #5, and #7 incorporated the neck residues of monomers, the original extensive connection network mediated by neck residues as an intramolecular “hub” in the presence of GDP no longer persisted. In the meantime, only five out of eight communities were still composed of residues from both monomers (Table S4), indicating weakened inter-monomeric crosstalk, which might facilitate the de-dimerization process of ROCs upon GTP binding to facilitate its activation.

### 2.5. Molecular Basis and Allostery Underlying Pathogenetic Effects of R1441C/G/H Mutations

Belonging to the catalytic core of LRRK2, the ROC domain represents a hotspot for PD-relevant pathogenic mutations. Residue R1441, located at the dimerization interface of ROCs homodimer, engages in the exquisite interaction network to stabilize the complex. Moreover, mutations of R1441 (R1441C/G/H) have been proven to be disease-causing based on segregation with disease in PD families [30,56,57,58]. To understand the detailed mechanism accounting for pathogenic effects induced by R1441 point mutation, we mutated the arginine at position 1441 to cysteine, glycine, and histidine, respectively, in the starting structure of ROCs-GDP, and obtained the ROCs*^R1441C^*-GDP, ROCs*^R1441G^*-GDP, and ROCs*^R1441H^*-GDP models for further investigations. The three systems were also subjected to large-scale MD simulation as that for ROCs-GDP/ROCs-GTP, gathering 3 μs extensive sampling for each mutated model. To probe into the disruption caused by R1441 mutations on the original conformational dynamics during nucleotide shift, we constructed kinetic MSM based on the data obtained with the same dimensionality-reduction transforming matrix and 3-ns lag time used for building ROCs-GDP~ROCs-GTP MSM, and yielded ROCs*^R1441C/G/H^*-GDP~ROCs-GTP MSMs with validated Markovianity, which could represent the conformational transition coupled with the activation of ROC homodimer under a mutated state (detailed in Section 4).

**Figure 5:**
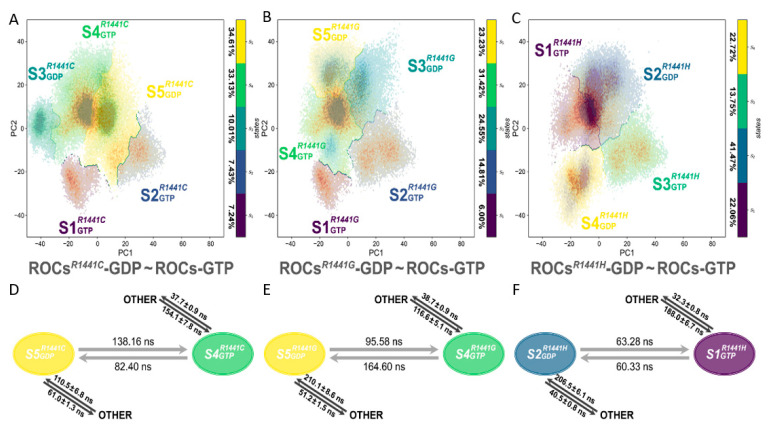
Distribution of metastable states in (**A**) ROCs*^R1441C^*-GDP~ROCs-GTP (5 macrostates), (**B**) ROCs*^R1441G^*-GDP~ROCs-GTP (5 macrostates), and (**C**) ROCs*^R1441H^*-GDP~ROCs-GTP (4 macrostates) MSM. The type of nucleotide ligand for ROCs is indicated with subscripts, and the percentage of each macrostate is shown beside the corresponding colormap. MFPT between the predominant GDP-bound ROCs mutant and GTP-bound ROCs calculated in (**D**) ROCs*^R1441C^*-GDP~ROCs-GTP, (**E**) ROCs*^R1441G^*-GDP~ROCs-GTP, and (**F**) ROCs*^R1441H^*-GDP~ROCs-GTP MSM. The two-ended black arrow between metastables and OTHER denoted the transition between the implicated macrostates and all other remaining metastable states in the corresponding MSM.

The whole conformational space was divided into 5, 5, and 4 metastables, respectively, for ROCs*^R1441C/G/H^*-GDP~ROCs-GTP MSMs (Figure 5A–C and Figure S4B–D) with a passing Chapman-Kolmogorov test (Figures S5–S7). In each MSM, based on the distribution pattern and fraction of the implicated systems in each metastable (Figure S4B–D, Table S1), we identified the most representative conformation for GDP-bound ROCs mutant and GTP-bound ROCs: *S5_GDP_^R1441C^* and *S4_GTP_^R1441C^* for the 5-metastable ROCs*^R1441C^*-GDP~ROCs-GTP MSM, *S5_GDP_^R1441G^* and *S4_GTP_^R1441G^* for the 5-metastable ROCs*^R1441G^*-GDP~ROCs-GTP MSM, *S2_GDP_^R1441H^* and *S1_GTP_^R1441H^* for the 4-metastable ROCs*^R1441H^*-GDP~ROCs-GTP MSM. The mutated ROCs*^R1441C/G/H^*-GDP shared much more overlap with ROCs-GTP on the PC1-PC2 plane (Figure 5A–C). MFPT was employed to estimate the relative conformational transition time between the GDP-bound ROCs mutant and GTP-bound ROCs (Figure 5D–F), and the results showed that mutation R1441C/G/H enabled a speeded shift from a GDP-bound inactive state to a GTP-bound active state (138.16, 95.58, and 63.28 ns compared with 143.39 ns) while also prominently accelerating the reverse transition (82.40, 164.60, and 60.33 ns compared with 446.58 ns). This observation supported the notion that R1441C/G/H mutation would result in overactivated ROCs, as this ROCs mutant could achieve faster conformational transition from a GDP-bound inactive state toward the GTP-bound active state. Moreover, inspection along the dimerization interface also disclosed the reduced interfacial area and disrupted bonding network mainly by the loss of the positively-charged R1441 sidechain upon mutation (Table S3). In the meantime, when measuring the dimerization binding free energy between monomers of these ROCs mutants with MM/PBSA (Table 1), a ~20 kcal/mol decrease was observed, demonstrating in energetic terms that ROCs harboring R1441C/G/H mutations were less dimerized and thus provided the basis for a faster transition toward the partially de-dimerized GTP-bound active state.

R1441 resides in α3 of the body subdomain in both monomer, far from structural motifs mediating nucleotide binding. While the effects of R1441 mutations on ROC dimerization seemed relatively straightforward (through disrupting interaction network), how this point mutation altered the dynamics of distant regions, especially motifs associated with GDP/GTP binding, remained elusive. To address this problem, we employed GCCM and visualized the intramolecular correlation pattern in the mutated systems (Figure S8). We found that, compared with the wild-type ROCs dimer (Figure 4A), all mutants exhibited attenuated correlation, while the weakening of coupled dynamics mediated by the body subdomain where the mutation rests was particularly obvious. This proved that R1441C/G/H mutations altered the global conformational landscape through reshaping the intramolecular communication pattern. Together with the observed disruption of dimerization, it could thus be speculated that mutations at position R1441 might result in a conformational shift of the ROC’s dimer toward certain states with a lower barrier to transmit into the GTP-bound active state [30].

**Figure 6:**
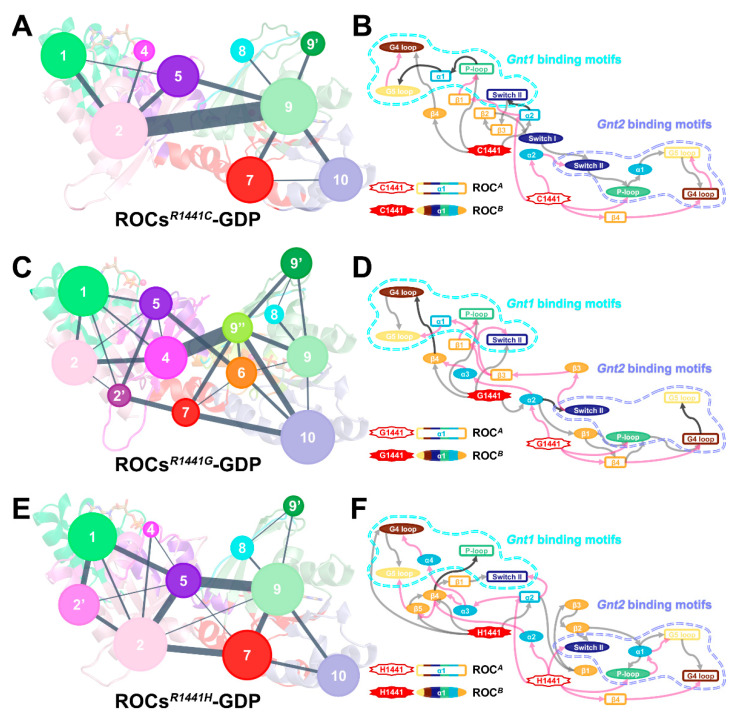
Simplified community network map with inter-community crosstalk in (**A**) ROCs*^R1441C^*-GDP, (**C**) ROCs*^R144G^*-GDP, and (**E**) ROCs*^R144H^*-GDP. Cartoon diagrams with residues color-coded by their corresponding community partition were made transparent as the background to indicate the spatial position of each community. A schematic diagram of allosteric signaling between the mutated site at position 1441 and the nucleotide-binding motifs *Gnt1* and *Gnt2* (marked with cyan and orchid dotted lines) in (**B**) ROCs*^R1441C^*-GDP, (**D**) ROCs*^R144G^*-GDP, and (**F**) ROCs*^R144H^*-GDP mutants. Secondary structures of ROC*^A^* are represented by colored hollow rectangles, and ROC*^B^* components are shown in filled ovals. The gray and pink arrows, respectively, indicate allosteric signals from the mutation site on ROC*^A^* and ROC*^B^*, and black arrows denote paths involved in transmitting signals from both mutation sites. Note that all arrows here are one-ended for clarity; the actual information flow can pass in both directions for reversible communication.

The global impact induced by R1441C/G/H residue substitutions suggested that the mutations could act through an allosteric mechanism. Therefore, CNA was performed to gain valuable insights into the conformational reorganization and allosteric signaling upon mutation. Although the community structures of the three mutants have their own distinctive features (Figure 6A,C,E), one commonality was the uniformly weakened inter-community crosstalk, leaving relatively strong information flow only between communities from the non-body subdomains (*Community* #2 and *Community* #9 in ROCs*^R1441C^*-GDP, *Community* #4 and *Community* #9 in ROCs*^R1441G^*-GDP, and *Community* #5 and *Community* #9 in ROCs*^R1441H^*-GDP; community composition is detailed in Table S4). We subsequently analyzed the allosteric signaling path communicating mutation site and the nucleotide-binding regions with the *subopt* program from *Dynamic Network Analysis* [59,60,61] and visualized the results in terms of secondary structure (Figure 6B,D,F). Our results showed that the neck subdomain (mainly α2) of both monomers was indispensable for transmitting signals induced by residue substitutions at R1441; meanwhile, this region was also implicated in propagating allosteric signals between monomers. Additionally, β4 from the body part of both monomers played a pivotal role in transmitting mutation-relevant allosteric signals within each monomer. In wild-type systems (Figure S9), crosstalk between ROC*^A^* α2 (neck) and ROC*^B^* β3 (head) bridged the functional units (ROCs1 and ROCs2) together in ROCs-GDP, while this signaling path was restructured upon GTP binding, with more segments participating in connecting the two functional units. For the mutants, the R1441C/G/H substitution also induced a change in signaling between functional units, as motifs from the body subdomain became directly involved in this process to enable a strengthened communication channel between distal regions, resembling the scheme observed in ROCs-GTP to some extent. In the meantime, the signaling network for ROCs*^R1441C/G/H^*-GDP is more concise with fewer roundabouts, indicating more efficient communication of allosteric signals from position 1441 to nucleotide-binding segments in mutants. Taken together, pathogenetic mutations R1441C/G/H acted through an allosteric mechanism and resulted in a global conformational shift of ROCs’ homodimer, which could enable a faster transition of the ROCs’ mutant toward the active state.

## 3. Discussion

Mutations of LRRK2 are recognized as the most common genetic cause of PD. While a plethora of pathogenic mutations are found within the ROC domain, considerably less attention has been paid to understanding the importance of GTPase activity in regulating LRRK2 function, and we do not know anything regarding the regulation of the ROC GTPase cycle associated with activation [14]. While existing conventional experimental approaches failed to illuminate the dynamic machinery, large-scale MD simulations can provide invaluable insight into the originally unreachable dynamic process when integrating with MSMs. In the present study, based on MSMs, we aimed to unveil the nature of conformational transition of the ROCs’ homodimer during GDP/GTP nucleotide turnover as well as quantitatively characterize the thermodynamic and kinetic properties. Through constructing ROCs-GDP~ROCs-GTP interconversion MSMs, we found that the ROCs’ dimer exhibited a differed dimerization extent in the presence of GDP/GTP, which resembled the nucleotide-dependent oligomerization and activation of LRRK2 homologue *Chlorobium tepidum* Roco protein, and further supported the proposed regulation of ROC GTPase cycle through nucleotide-dependent dimerization. Interestingly, when estimating the relative transition time between inactive GDP-bound and active GTP-bound state, we found that the transition from ROCs-GDP to ROCs-GTP took only approximately 1/3 of the time needed for the reverse process, and this difference suggested the need for GTPase-activating proteins (GAP) that accelerate hydrolysis and render the complex inactive. Indeed, GAP-like proteins for LRRK2, as exemplified by ArfGAP1, have been reported [24,52,62]. The coupling between dimerization extent and nucleotide-binding state was further demonstrated by the observed conformational shift resembling the classic “open” to “closed” structural rearrangements observed for typical small GTPase like Ras [38], with regions implicated in nucleotide binding such as P-loop, Switch II, G4, and G5 loop, forming hydrogen bonds with the incoming γ-phosphate and thus yielding the “closed” conformation under a GTP-bound state. Probing into the correlation network that mediates the observed shift in conformational landscape, we found prominently attenuated inter-monomeric crosstalk upon GTP binding based on GCCM analysis, while a reshaped community structure with weakened signaling also suggested the potential oligomerization of ROCs-GTP during its GTPase cycle. On the whole, our present studies based on extensive MD simulations and MSMs suggested that the ROC domain of LRRK2 is likely to function through a nucleotide-dependent dimerization-based activation manner, similar to the GADs.

With our established activation model, we further investigated the mechanism underlying the pathogenic effects of missense mutations at R1441. A point mutation at this site was found to transform the intramolecular correlation pattern and community structure, resulting in faster conformational interconversion between the GDP-bound inactive and GTP-bound active state for a mutated ROCs homodimer. While previous studies have proved that R1441C/G/H mutations can both increase binding affinity for GTP and decreas GTPase activity to varying degrees, our present study expounded from the previously-unnoted aspect that such mutations were also likely to speed up the conformational transition during the GTPase activation cycle to trap ROCs in a more persistently active state, which would ultimately contribute to LRRK2 dysfunction and PD pathogenesis. In light of the effects of R1441C/G/H point mutations on the global conformational landscape and bioactivity of ROCs, we presumed that mutation-induced allostery played a key role in altering interdependent conformational dynamics within ROCs. Therefore, with allosteric signaling pathway analysis, structural motifs implicated in transmitting mutation-relevant allosteric signals were identified, which suggested the existence of potential allosteric pockets on the ROCs’ homodimer for regulating its functionality. Taken together, the present study illuminated the dynamic conformational transition, which is coupled with nucleotide binding of the ROC domain of LRRK2, as a prototype for the ROCO proteins, during the GTPase activation cycle. Moreover, inspired by the disease-causing mutations within this domain, as exemplified by R1441C/G/H point mutations, we revealed the underlying allostery of pathogenic effects, which would provide the basis for targeting such an allosteric mechanism to enable rational control of ROCs’ bioactivity in both physiological and pathological contexts for PD treatment.

## 4. Materials and Methods

### 4.1. Construction of Simulation Systems

The five simulation systems (wildtype: ROCs-GDP and ROCs-GTP; mutant: ROCs*^R1441C^*-GDP, ROCs*^R1441G^*-GDP, and ROCs*^R1441H^*-GDP) in our study were built on the basis of the solved crystal structure of ROC homodimer in complex with GDP (PDB ID: 2ZEJ) [20]. The missing residues (T1357-D1387 and K1512-Q1516) in the original crystal profile were re-modeled with *Discovery Studio* [63], followed by subjecting the whole system to two rounds of 5000-step minimization using the steepest descent algorithm typed with a CHARMM forcefield [64]. The GTP-bound ROCs were obtained through introducing a γ-phosphate on the original GDP, and minimization was conducted on regions near the two nucleotide-binding sites to avoid potential structural conflicts. For the mutated systems, the arginine at position 1441 of both monomers in ROCs-GDP was mutated to cysteine (ROCs*^R1441C^*-GDP), glycine (ROCs*^R1441G^*-GDP), and histidine (ROCs*^R1441H^*-GDP), respectively.

### 4.2. MD Simulations

The parameter files for minimization and simulation were prepared with the Amber18 package using the Amber ff14SB force field and general amber force field (GAFF) [65,66]. All systems were first solvated in an orthorhombic transferable intermolecular potential three-point (TIP3P) water box [67], and Na^+^ and Cl^−^ counterions were subsequently added to attain the physiological saline concentration. Energy minimization was performed on all systems using the steepest descent and conjugate gradient algorithm [68,69] with protein scaffold fixed for 10 ps and without any constraint for 20 ps, respectively. All five systems were then put in a canonical ensemble (NVT) and were heated from 0 to 300 K within 300 ps with all protein atoms constrained, followed by being subjected to a 700 ps equilibration run. Finally, a total of 3 μs (1 × 3 μs replica) conventional all-atom MD simulations was conducted on each system in an isothermal and isobaric ensemble (NPT) with periodic boundaries. Langevin dynamics using 1 ps^−1^ collision frequency was chosen for the temperature control [70]. The Particle Mesh Ewald (PME) method was employed to analyze long-range electrostatic interactions [71], while a 10 Å cutoff was selected to treat short-range electrostatics and van der Waals forces. The SHAKE algorithm was used to constrain bond interactions involving hydrogens. The trajectory information of all systems was collected every 50 ps in each productive run.

### 4.3. Markov State Model Construction and Validation

Traditionally, to investigate the comprehensive conformational rearrangements in a dynamic context, large-scale MD simulations with few/no replications were often applied [72,73,74]. Such extensive sampling could allow access to slow dynamics, which often occur on a larger timescale and have been indicated to be responsible for intramolecular allosteric signaling [36,75]. However, due to the forbidding expense required to conduct such extensive simulations, the reproducibility of such work should be carefully assessed considering the lack of replications. In the meantime, integrating Markov state modeling (MSM) with MD simulations is gaining increasing popularity for the efficiency and accuracy that can be reached when interpreting biomolecular dynamics, and this combination has been proven reproducible when verifying with experimental techniques [22,39]. Nonetheless, it is clear that with very short trajectories, the intramolecular correlation network and also other important characteristics presented during the time-consuming conversion process would thus be uninvestigable. Under such circumstances, we constructed our statistical MSM using high-dimensional time-series data from several extensive simulations (3 × 1 μs), and this innovative scheme could allow the combination of the advantages of the aforementioned methodologies, achieving both the detailed atomistic insights of traditional extensive simulations and statistical accuracy by integrating MSM only with short simulations.

We employed the Python library *PyEMMA* for the estimation, validation, and analysis of Markov state models (MSM) from MD data [42]. To understand the kinetics and thermodynamics of ROCs during nucleotide turnover under physiological conditions, we first built an MSM based on the simulation data from ROCs-GDP and ROCs-GTP systems (ROCs-GDP~ROCs-GTP MSM) to probe into their interconversion. First, the raw Cartesian data were featurized with inter-residue (represented by Cα atoms) distances to describe the overall ROCs topology [45]. Subsequently, with the aim of capturing slow dynamics and primary conformational changes, we reduced the complexity of our data by carrying out dimensionality reduction with principal component analysis embedded in the *scikit-learn* python package [43,45,76,77]. Of note, we recorded the transforming matrix here for further application. Through implied timescales analysis, we validated the Markovianity of our data when discretized with 100 microstate cluster centers (Figure S2). After careful selection, a lag time of 3 ns and the maximum 200 *k-means* iteration number with 100 centers were chosen to cluster the data into microstates. The obtained microstates were then lumped into four metastable states using the PCCA+ algorithm with a passing Chapman-Kolmogorov test [78] (Figure S3). Transition pathway theory (TPT) was employed to measure the transition probability matrix of the constructed MSM [47,48,79] as well as compute the mean first passage time (MFPT) between selected states. The MSM describing kinetic interconversion between mutated ROCs*^R1441C/G/H^*-GDP and ROCs-GTP were also constructed in the same workflow with the recorded transforming matrix used for ROCs-GDP ~ROCs-GTP dimensionality reduction also employed here for the same purpose. Implied timescales and a Chapman-Kolmogorov test were also used to validate the Markovianity of the constructed models (Figures S2 and S5–S7).

### 4.4. Generalized Cross-Correlation Matrix Analysis

Generalized cross-correlation matrix (GCCM) analysis, proposed by Grubmüller and Lange [53], was employed in our study for the assessment of both linear and nonlinear correlated motions. GCCM adopted the fundamental definition of independence of random variables, and treated variables $x_{i},\;x_{j}$ correlated only when the product of their marginal distribution $p(x_{i})\cdot p(x_{j})$ is larger than their joint distribution $p(x_{i},x_{j})$. Thus, mutual information (*MI*) between $x_{i}$ and $x_{j}\;$ is defined as:

(1) $$MI\left[x_{i},x_{j}\right]=\iint \;p\left(x_{i},x_{j}\right)ln\frac{p(x_{i},x_{j})}{p(x_{i})\cdot p(x_{j})}dx_{i}dx_{j}$$

which measures the degree of correlation between selected variables. Notably, the right part of Equation (1) is closely related to the well-known Shannon entropy H[x] [80], which can be obtained through:

(2) H[x]=∫ p(x)lnp(x)dx

Therefore, the correlation between pairs of atoms $x_{i}$ and $x_{j}$ can be described with *MI* and calculated through the marginal Shannon entropy $H\left[x_{i}\right]$, $H\left[x_{j}\right]$, together with the joint entropy term $H\left[x_{i},x_{j}\right]$ through:

(3) $$MI\left[x_{i},x_{j}\right]=H\left[x_{i}\right]+H\left[x_{j}\right]-H\left[x_{i},x_{j}\right]$$

where the entropy terms $H\left[x_{i}\right]$, $H\left[x_{j}\right]$, and $H\left[x_{i},x_{j}\right]$ can be calculated with the *g_correlation* tool in Gromacs 3.3 [81]. The final generalized correlation coefficients ($GC_{ij}$) can be obtained through normalizing $MI\left[x_{i},x_{j}\right]$ with:

(4) $$GC_{ij}={\left\{1-e^{-\frac{2MI\left[x_{i},x_{j}\right]}{d}}\right\}}^{\frac{1}{2}}$$

In which *d* represents the dimensionality of $x_{i}$ and $x_{j}$. In our studies, *d* = 3.

### 4.5. Dynamic Network Analysis

Concepts from network theories [41,82] were employed to investigate the residue–residue interaction network [41]. The whole ROCs homodimer of each system (except nucleotide and Mg^2+^ ion) was treated as a set of nodes (assigned to the Cα atom of each residue) connected through edges, which would be drawn between nodes that stayed within a proximity of 4.5 Å for at least 75% of the simulation time. To indicate the relative distance for information transfer through a certain edge between nodes i and j, $GC_{ij}$ coefficients were used to weight the edges through:

(5) $$d_{ij}=-logGC_{ij}$$

The obtained $GC_{ij}$-weighted correlation network provides a basis for further community network analysis [54] (CNA) and allosteric pathway analysis [59,60,61]. The community substructure was obtained with the *gncommunities* program, which embedded the Girvan-ewman divisive algorithm and used “edge betweenness” that defines the number of shortest pathways across a given edge as an important partition criterion. To obtain the optimal substructure of the network, edges with the highest betweenness would be iteratively removed from the network, and the remaining ones would be recalculated until each node represents an isolated community.

The Floyd–Warshall algorithm [83] was used to identify the “shortest pathway” between selected pairs of nodes (defined as source and sink) by comparing the sum of $d_{ij}$ of all edges implicated in the paths. In our studies, we treat R1441/C1441/G1441/H1441 as the source, while sinks were laid in regions associated with nucleotide binding, with G1346 representing P-loop, D1394 representing Switch II, D1455 representing G4 loop, and A1490 representing G5 loop.

## Figures and Tables

**Table 1:** ROC monomers MM/PBSA-binding free energy (kcal/mol) *.

Systems	Representative	Binding Free Energy	Standard Deviations
ROCs-GDP	*S3_GDP_*	−296.39	19.71
ROCs-GTP	*S2_GTP_*	−285.09	23.52
ROCs*^R1441C^*-GDP	*S5_GDP_^R1441C^*	−276.31	18.02
ROCs*^R1441G^*-GDP	*S5_GDP_^R144G^*	−274.93	17.48
ROCs*^R1441H^*-GDP	*S2_GDP_^R144H^*	−274.98	17.64

* The MM/PBSA-binding free energies between ROC monomers serve as an indicator for ROC dimerization extent.

## Data Availability

Samples of the simulation trajectories are available from the authors upon request.

## Supplementary Materials

The following are available online, Figure S1: Real-time root-mean-square deviations (RMSD) of all systems during simulation, Figure S2: Implied timescales estimation of all constructed MSMs, Figure S3: Results of Chapman-Kolmogorov test when assuming 4 metastable states in ROCs-GDP~ROCs-GTP MSM, Figure S4: Distribution of the metastable states in all constructed MSMs, Figure S5: Results of Chapman-Kolmogorov test when assuming 5 metastable states in ROCs-GDPR1441C~ROCs-GTP MSM, Figure S6: Results of Chapman-Kolmogorov test when assuming 5 metastable states in ROCs-GDPR1441G~ROCs-GTP MSM, Figure S7: Results of Chapman-Kolmogorov test when assuming 5 metastable states in ROCs-GDPR1441H~ROCs-GTP MSM, Figure S8: GCCM matrix for mutated systems, Figure S9: Allosteric signaling path between R1441 and nucleotide binding regions in wildtype systems, Table S1: Percentage of each metastable structure in each MSM, Table S2: Bonding network formed between ROC monomers in ROCs-GDP and ROCs-GTP, Table S3: Interfacing area (Å2) and number of stable bonds for representative structures in each system, Table S4: Residue compositions of the identified communities in each system.

## Sample Availability

Samples of the intermediate structures mentioned in this work are available from the authors upon request.
