# Supplementary material for: Markov State Models and Molecular Dynamics Simulations Provide Understanding of the Nucleotide-Dependent Dimerization-Based Activation of LRRK2 ROC Domain
Source: Molecules. 2021 Sep 17;26(18):5647. doi: 10.3390/molecules26185647 (PMC8467336; doi:10.3390/molecules26185647)
Supplement: Supplementary file 1 [file molecules-26-05647-s001.zip › molecules-1370266-supplementary.pdf]

Supplementary Figures

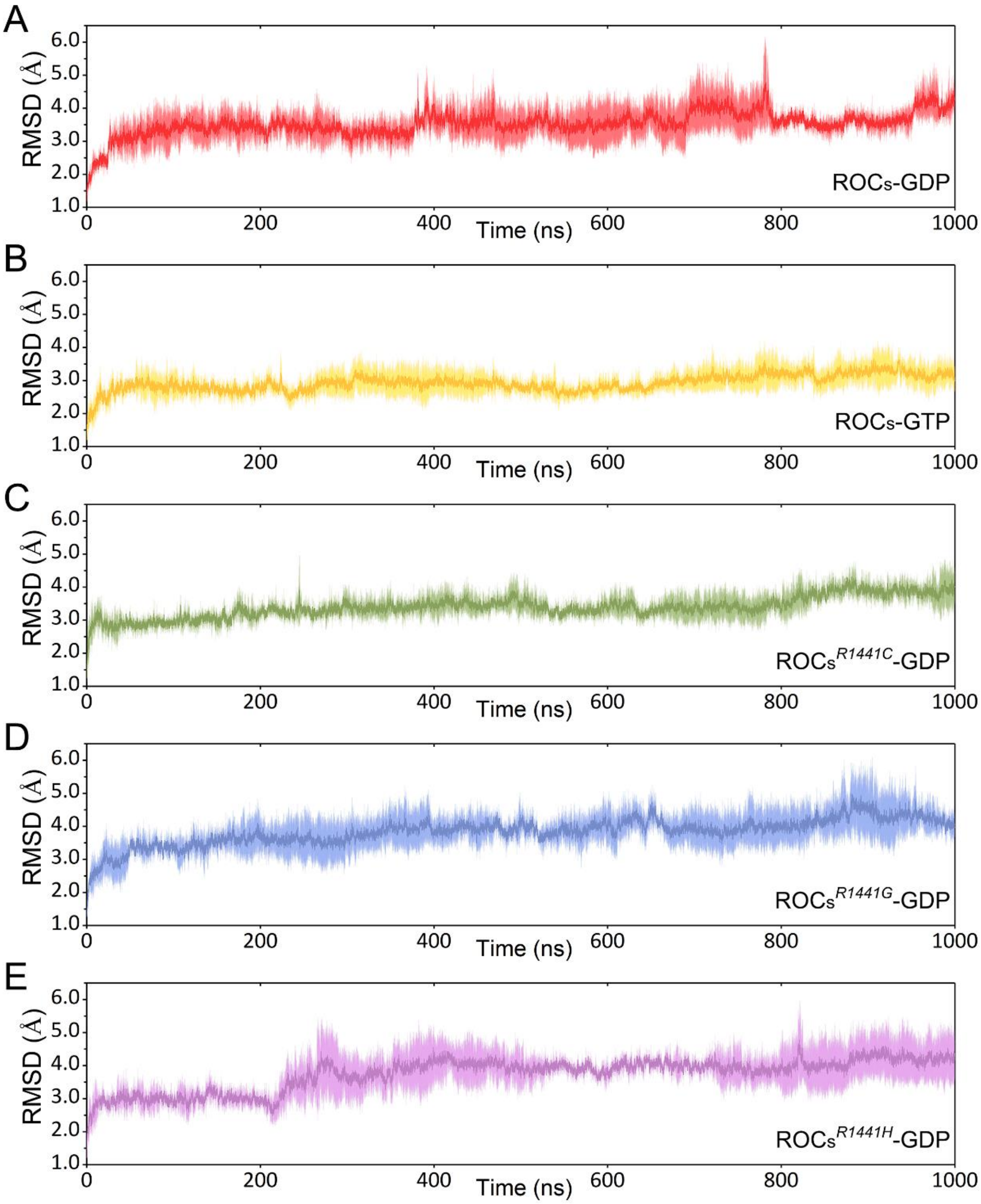

Figure S1 Real-time root-mean-square deviations averaged over 3 replication runs in (A) ROCs-GDP (red), (B) ROCs-GTP (yellow), (C) ROCs^{R1441C}-GDP (green), (D) ROCs^{R1441G}-GDP (blue) and (E) ROCs^{R1441H}-GDP (orchid). RMSD were calculated using backbone atoms. The opaque line depicted the average RMSD value, the vertical transparent dash indicated standard deviations.

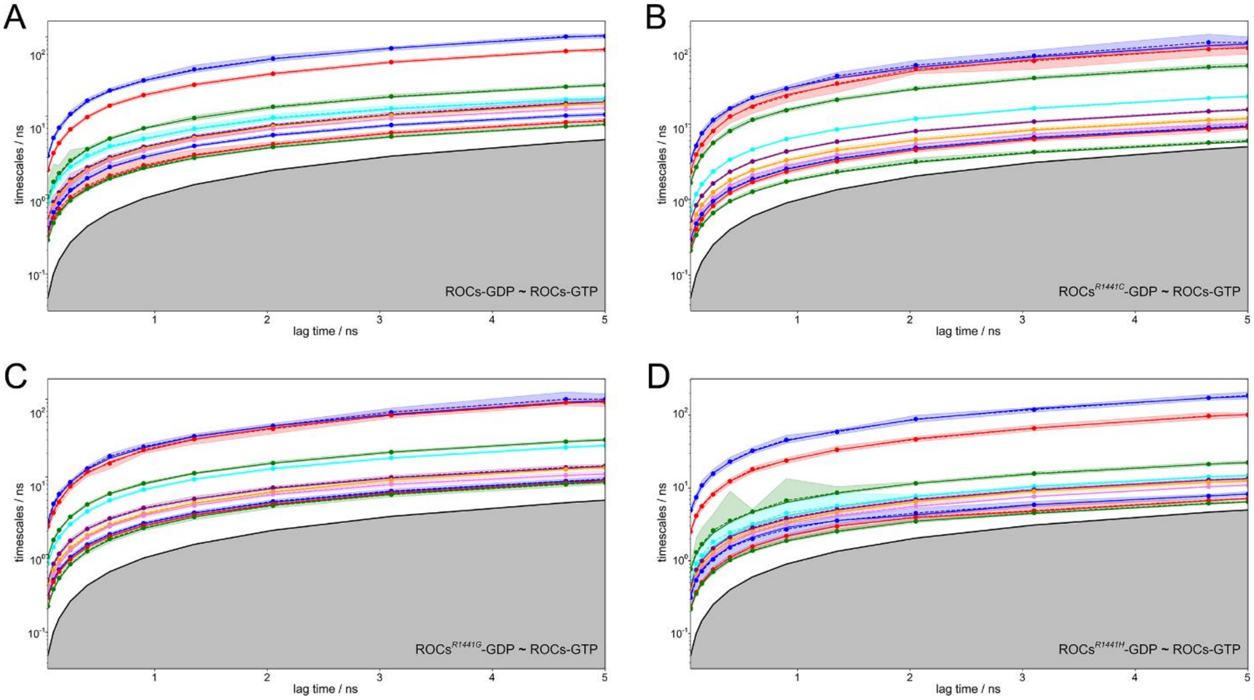

Figure S2 Implied timescales estimation of MSM built with trajectory information from (A) ROCs-GDP and ROCs-GTP (ROCs-GDP ~ ROCs-GTP), (B) ROCs^{R1441C}-GDP and ROCs-GTP (ROCs^{R1441C}-GDP ~ ROCs-GTP), (C) ROCs^{R1441G}-GDP and ROCs-GTP (ROCs^{R1441G}-GDP ~ ROCs-GTP), (D) ROCs^{R1441H}-GDP and ROCs-GTP (ROCs^{R1441H}-GDP ~ ROCs-GTP). Implied timescales of maximum likelihood MSM is depicted in solid lines, while dashed line denoted sample mean, and the shaded area are 95% confidential intervals from Bayesian MSM. From the above implied timescales result that converged quickly, we chose a lag time of 3 ns to build our Markov models.

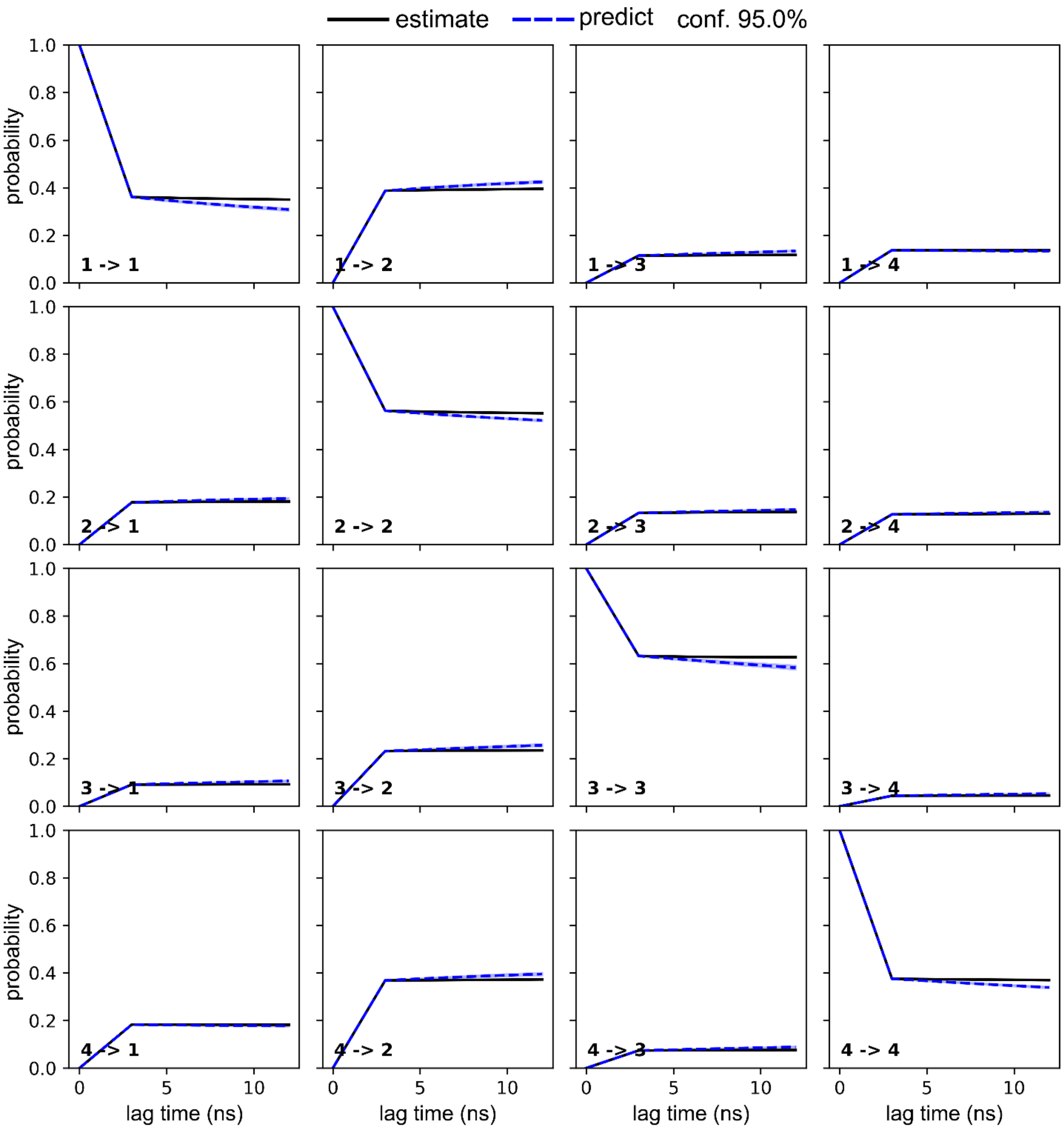

Figure S3 Results of Chapman-Kolmogorov test when assuming 4 metastable states in ROCs-GDP ~ ROCs-GTP MSM.

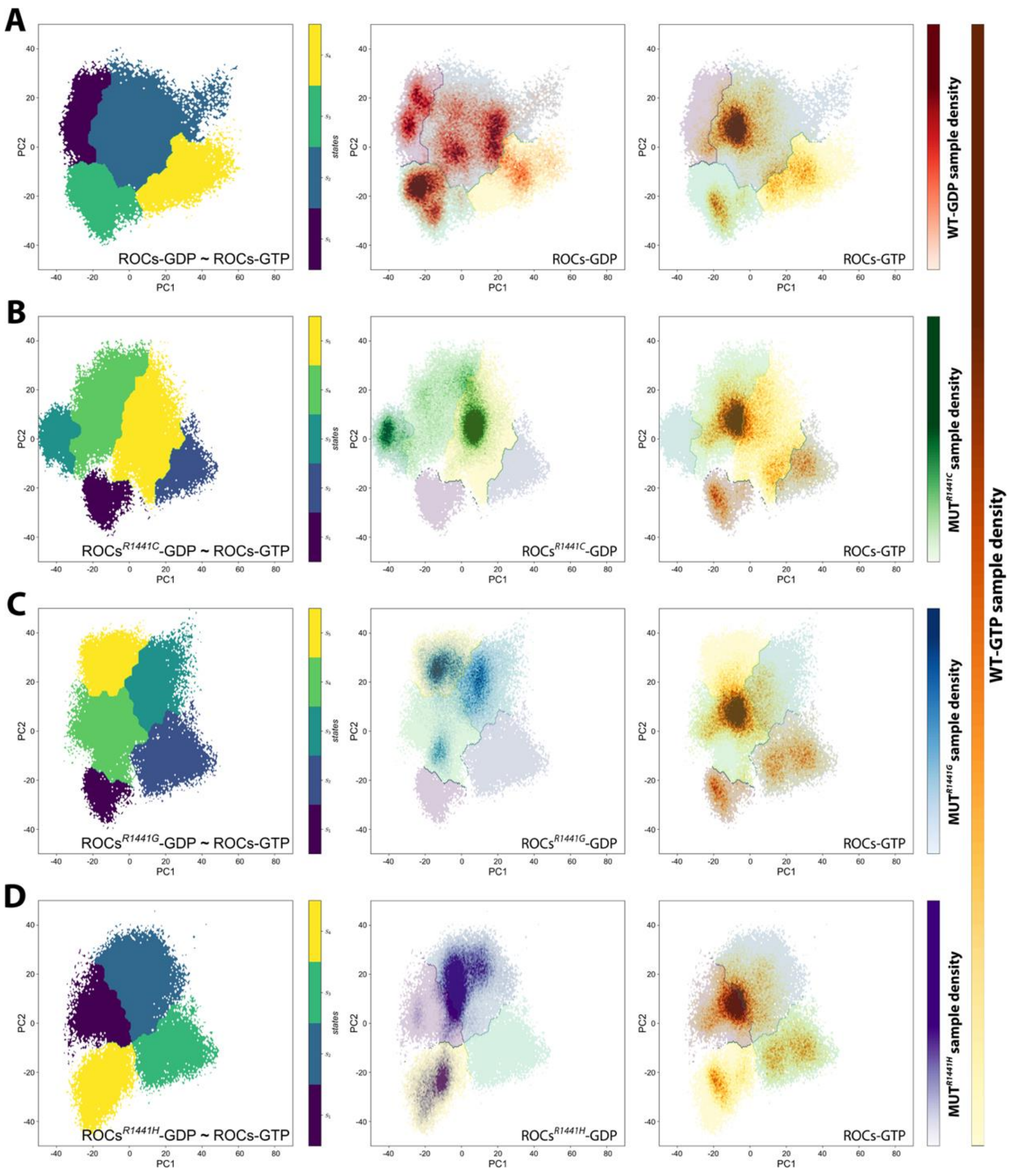

Figure S4 Distribution of the metastable states of MSM in (A) ROCs-GDP ~ ROCs-GTP, (B) ROCs^{R1441C}-GDP ~ ROCs-GTP, (C) ROCs^{R1441G}-GDP ~ ROCs-GTP, (D) ROCs^{R1441H}-GDP ~ ROCs-GTP. The first column illustrated location of the macrostates on the PC1-PC2 plane, while the other columns indicated the distribution of each system among the metastable states in their corresponding MSM using density map. The colormaps are shown on the right (ROCs-GDP: red, ROCs-GTP: yellow, ROCs^{R1441C}-GDP: green, ROCs^{R1441G}-GDP: blue, ROCs^{R1441H}-GDP: orchid).

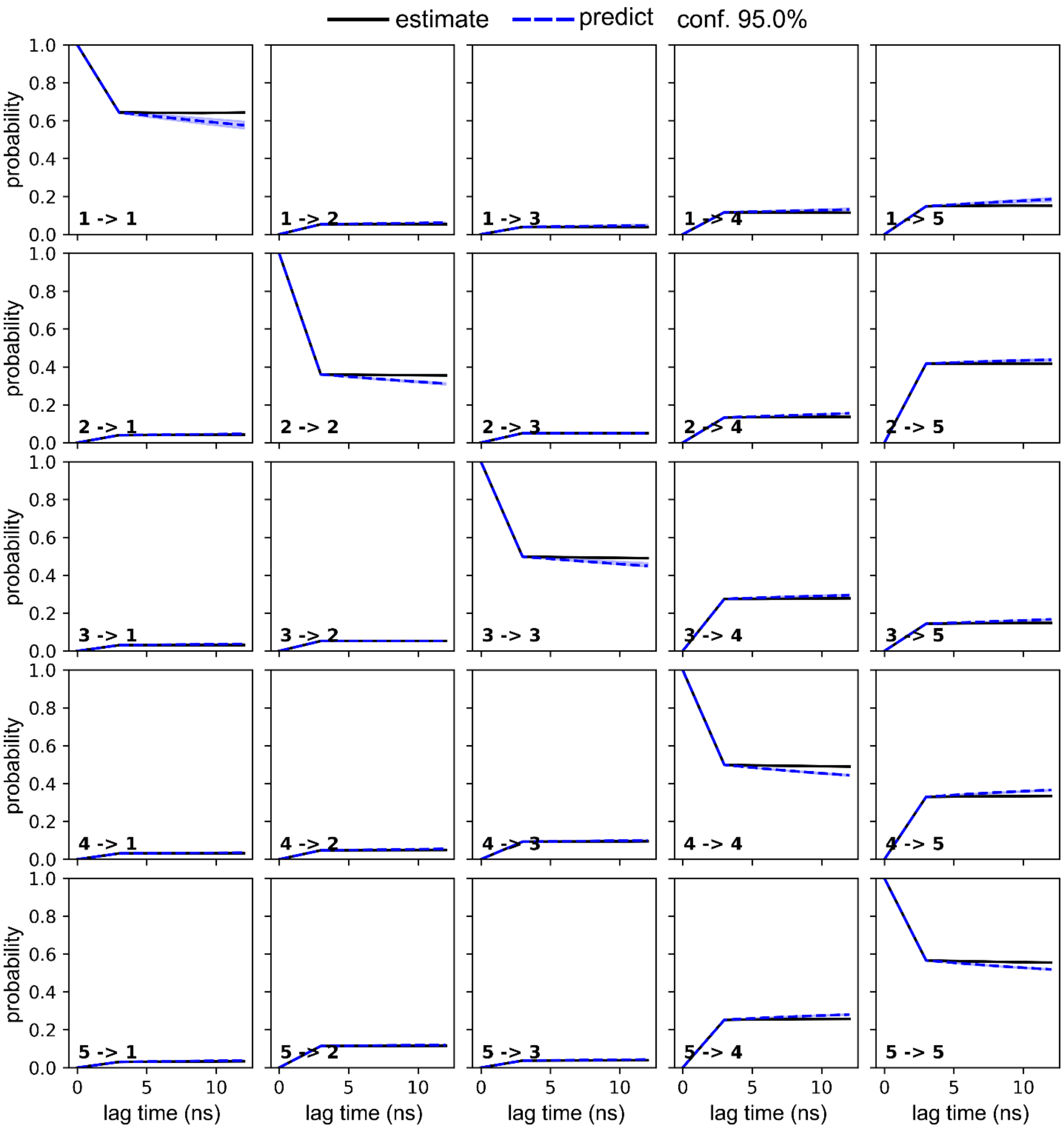

Figure S5 Results of Chapman-Kolmogorov test when assuming 5 metastable states in ROCs-GDP^{R1441C} ~ ROCs-GTP MSM.

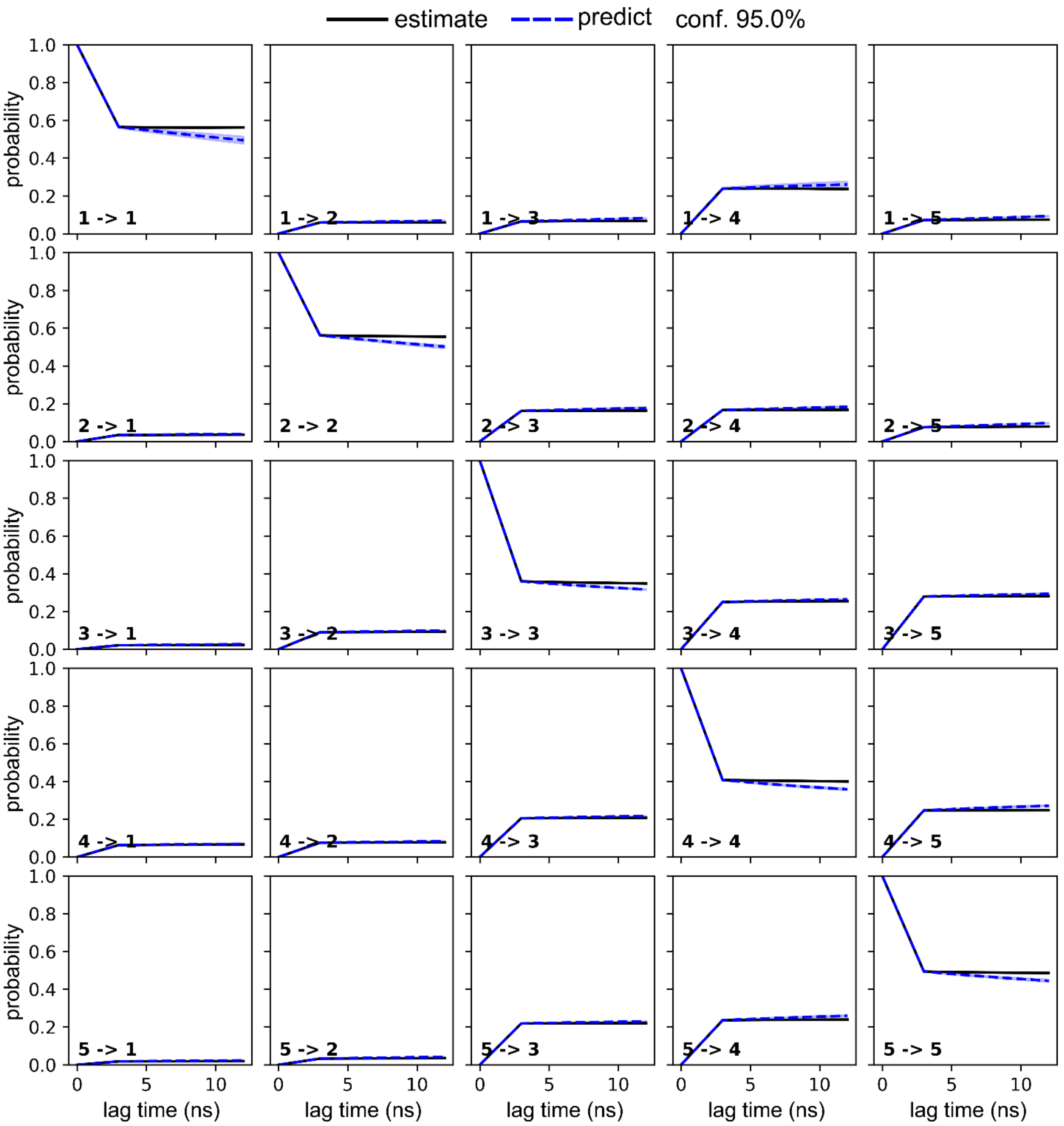

Figure S6 Results of Chapman-Kolmogorov test when assuming 5 metastable states in ROCs-GDP^{R1441G} ~ ROCs-GTP MSM.

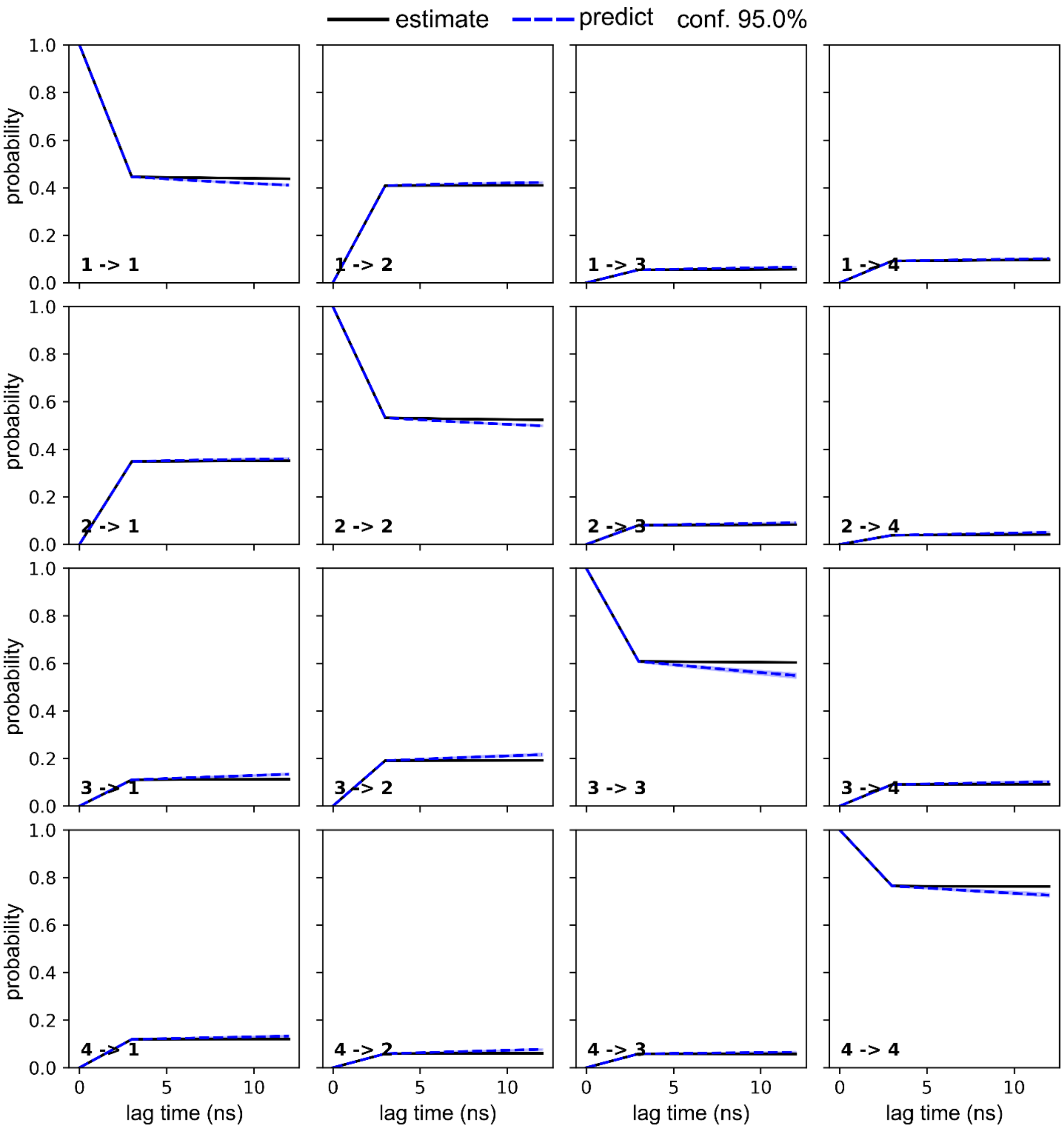

Figure S7 Results of Chapman-Kolmogorov test when assuming 4 metastable states in ROCs-GDP^{R1441H} ~ ROCs-GTP MSM.

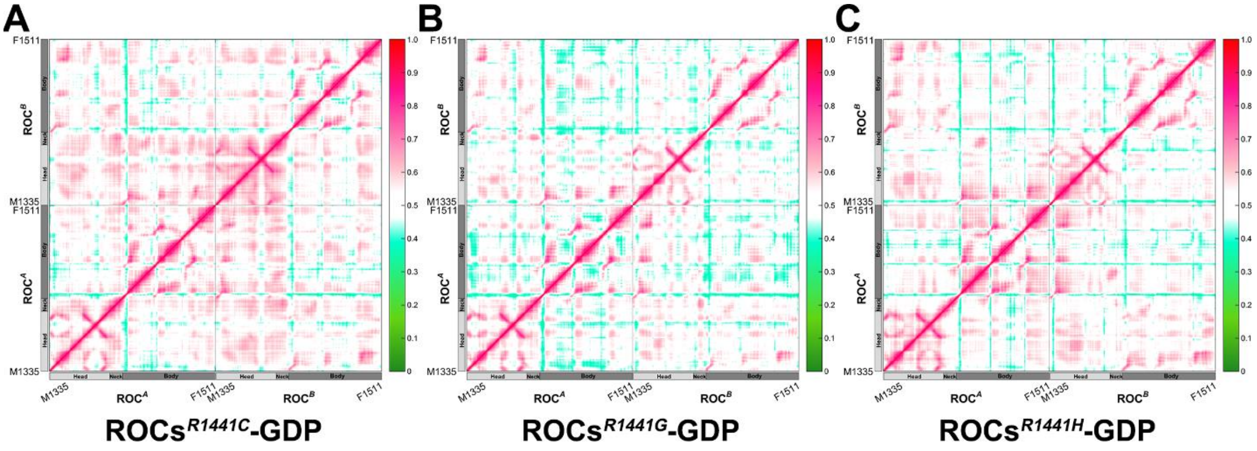

Figure S8 GCCM of (A) ROCs^{R1441C}-GDP, (B) ROCs^{R1441G}-GDP and (C) ROCs^{R1441H}-GDP. The colormap used for each graph are shown on the right with no/weak correlation indicated in green and strong ones in magenta.

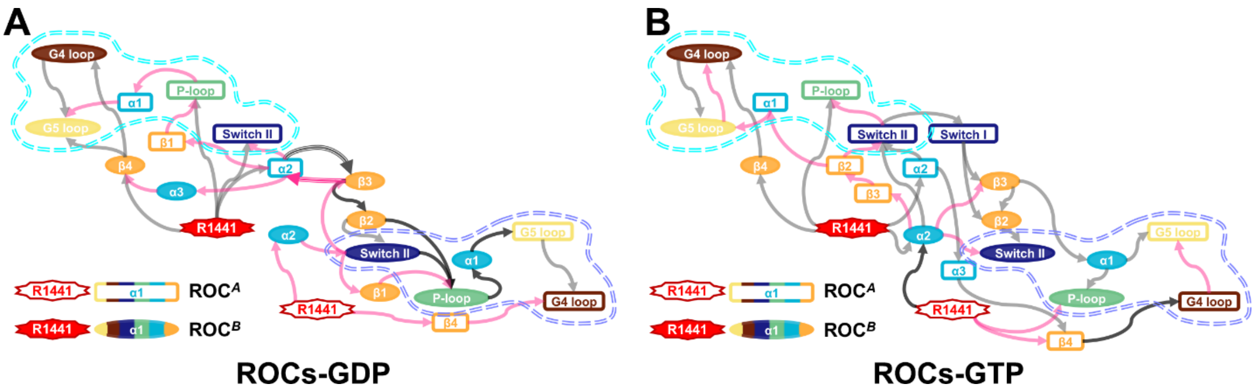

Figure S9 Schematic diagram of allosteric signaling between R1441 and the nucleotide binding motifs *Gnt1* and *Gnt2* (marked with cyan and orchid dotted lines) in (A) ROCs-GDP and (B) ROCs-GTP. Secondary structures of ROC^{A} are represented by colored hollow rectangles, and ROC^{B} components are shown in filled ovals. The gray and pink arrows respectively indicate allosteric signals from mutation site on ROC^{A} and ROC^{B}, and black arrows denoted paths involved in transmitting signals from both mutation sites. All arrows here are one-ended for clarity, the actual information flow can pass in both directions for reversible communication.

Supplementary Tables

Table S1 Percentage of each metastable structure in each MSM*.

- ROCs-GDP ~ ROCs-GTP MSM

	Overall	ROCs-GDP	ROCs-GTP
$S1_{GDP}$	9.62%	14.07%	6.58%
$S2_{GTP}$	53.70%	49.20%	62.34%
$S3_{GDP}$	20.15%	24.72%	11.50%
$S4_{GTP}$	16.53%	12.01%	19.58%

- ROCs-GDP^{R1441C} ~ ROCs-GTP MSM

	Overall	ROCs^{R1441C}-GDP	ROCs-GTP
$S1_{GTP}^{R1441C}$	7.24%	0.17%	10.96%
$S2_{GTP}^{R1441C}$	7.43%	0.41%	13.65%
$S3_{GDP}^{R1441C}$	10.01%	18.47%	0.84%
$S4_{GTP}^{R1441C}$	33.13%	29.38%	39.94%
$S5_{GDP}^{R1441C}$	42.19%	51.57%	34.61%

- ROCs-GDP^{R1441G} ~ ROCs-GTP MSM

	Overall	ROCs^{R1441G}-GDP	ROCs-GTP
$S1_{GTP}^{R1441G}$	6.00%	0.17%	9.84%
$S2_{GTP}^{R1441G}$	14.81%	1.02%	26.34%
$S3_{GDP}^{R1441G}$	24.55%	38.62%	12.06%
$S4_{GTP}^{R1441G}$	31.42%	20.29%	43.69%
$S5_{GDP}^{R1441G}$	23.23%	39.89%	8.06%

- ROCs-GDP^{R1441H} ~ ROCs-GTP MSM

	Overall	ROCs^{R1441H}-GDP	ROCs-GTP
$S1_{GTP}^{R1441H}$	22.06%	14.96%	32.32%
$S2_{GDP}^{R1441H}$	30.31%	59.09%	30.31%

$S3_{GTP}^{R1441H}$	25.42%	0.30%	25.42%
$S4_{GDP}^{R1441H}$	11.95%	25.64%	11.95%

*The nucleotide-binding state of each representative metastable structure is defined by the population and distribution of each system in the corresponding macrostates. The system with more prominent contribution in each metastable states is highlighted in gray.

Table S2 Bonding network formed between ROC monomers in ROCs-GDP and ROCs-GTP*

Roc^A	ROCs-GDP		ROCs-GTP		Roc^B
	Fraction[1]	Avg_dist[2]	Fraction	Avg_dist	
K1336_H	0.59	3.07	0.39	3.09	R1412_O
K1336_O	0.91	3.06	0.94	3.04	L1414_H
M1338_H	0.97	2.97	0.97	2.92	L1414_O
M1338_O	0.98	3.03	0.98	2.99	L1416_H
V1340_H	0.99	2.92	1	2.98	L1416_O
V1340_O	0.98	2.98	0.98	2.97	V1418_H
S1345_O	0.93	2.73	0.58	2.73	T1452_HG1
Q1353_O	0.63	2.87	0.58	2.86	R1501_HH21
L1354_O	0.67	2.94	0.61	2.95	R1501_HE
F1401_O	0.56	3	0.58	2.9	R1441_HH22
T1404_OG1	0.55	2.91	0.36	2.86	R1441_HH12
R1412_O	0.47	2.99	0.56	3.05	K1336_H
L1414_H	0.95	3.01	0.96	3.05	K1336_O
L1414_O	0.96	2.92	0.96	2.94	M1338_H
L1416_H	0.98	2.95	0.98	2.96	M1338_O
L1416_O	0.99	3.01	0.99	2.97	V1340_H
V1418_H	0.97	3.01	0.97	2.96	V1340_O
E1427_OE2	0.46	3.02	0.38	3.04	N1342_HD22
A1430_O	0.75	3.25	0.38	3.33	N1342_HD21
W1434_HE1	0.91	2.94	0.7	2.98	N1342_OD1
R1441_HH12	0.37	2.92	0.46	2.92	F1401_O
R1501_HH21	0.58	2.86	0.71	2.85	Q1353_O
R1501_HE	0.63	2.99	0.74	2.95	L1354_O

[1]Fraction: the fraction of frames with distance between the bonding atoms less than 3.5 Å;
[2]Avg_dist: the average distance between the bonding atoms.
*Atoms involved in bonding is indicated in the first and last columns. Bonding pairs with prominent different in two systems are highlighted in gray.

Table S3 Interfacing area (Å^2) and number of stable bonds (fraction > 60%, N_{stable_bonds}) for representative structures in each system*.

System	Representatives	Interfacing area	N_{stable_bonds}
ROCs-GDP	$S3_{GDP}$	3519.0	16
ROCs-GTP	$S2_{GTP}$	3365.8	14
ROCs^{R1441C}-GDP	$S5_{GDP}^{R1441C}$	3133.8	11
ROCs^{R1441G}-GDP	$S5_{GDP}^{R144G}$	3037.2	11
ROCs^{R1441H}-GDP	$S2_{GDP}^{R144H}$	3107.8	10

*The interfacing area are calculated with PDBePISA (Proteins, Interfaces, Structures and Assemblies); N_{stable_bonds} are calculated through scanning throughout all the trajectory and only count the bonds that exist in more than 60% of the frames.

Table S4 Residue compositions of the identified communities in each system.

- ROCs-GDP

Community	Compositions
1	A_Body:42, B_Head:3
2	B_Head:35, A_Body:33, B_Body:1
3	B_Head:8
4	B_Head:12
5	A_Body:24, B_Neck:7, B_Head:5, A_Neck:2
6	B_Neck:7, A_Head:2, B_Body:2
7	B_Body:18, A_Neck:12, A_Head:6
8	A_Head:13
9	A_Head:40, B_Body:29
10	B_Body:50, A_Head:3

- ROCs-GTP

Community	Compositions
1	A_Body:40
2	B_Head:53, A_Body:32, A_Neck:6, B_Body:1
3	B_Head:5
5	A_Body:27, B_Neck:14, B_Head:5
7	B_Body:27, A_Neck:8, A_Head:6
8	A_Head:14
9	A_Head:42, B_Body:30
10	B_Body:42, A_Head:2

- ROCs^{R1441C}-GTP

Community	Compositions
1	A_Body:38, B_Head:7
2	B_Head:40, A_Body:29, A_Neck:1, B_Body:1
4	B_Head:9
5	A_Body:32, B_Neck:8, B_Head:7, A_Neck:1
7	B_Body:32, A_Neck:12, A_Head:3
8	A_Head:11
9	A_Head:35, B_Body:29, B_Neck:6
9'	A_Head:12
10	B_Body:38, A_Head:3

- ROCs^{R1441G}-GTP

Community	Compositions
1	A_Body:48,B_Head:6
2	A_Body:29,B_Head:15
2'	A_Body:11
4	B_Head:38,B_Body:1
5	A_Body:11,B_Neck:8,B_Head:4
6	B_Body:11,B_Neck:6,A_Head:3
7	A_Neck:13,A_Head:3
8	A_Head:7
9	B_Body:27,A_Head:9
9'	A_Head:21
9''	A_Head:18,B_Body:1
11	B_Body:60,A_Head:3,A_Neck:1

- ROCs^{R1441H}-GTP

Community	Compositions
1	A_Body:45, B_Head:5
2	B_Head:34, A_Body:24, A_Neck:8, B_Body:1
2'	A_Body:19, B_Head:17
4	B_Head:5
5	B_Neck:13, A_Body:11, B_Head:2
7	B_Body:37, A_Head:10, A_Neck:6
8	A_Head:11
9	A_Head:34, B_Body:25, B_Neck:1
9'	A_Head::8
10	B_Body:37, A_Head:1

*The number of residues form head, neck or body portion of either monomer is shown here to indicate the residue composition for each monomer.
